# Lifetime prevalence and correlates of self-harm and suicide attempts among male prisoners with histories of injecting drug use

**DOI:** 10.1186/s40352-018-0077-2

**Published:** 2018-10-15

**Authors:** Ashleigh C. Stewart, Reece Cossar, Paul Dietze, Gregory Armstrong, Michael Curtis, Stuart A. Kinner, James R. P. Ogloff, Amy Kirwan, Mark Stoové

**Affiliations:** 1 Behaviours and Health Risks, Burnet Institute, Melbourne, VIC Australia; 2 School of Public Health and Preventative Medicine, Monash University, Melbourne, VIC Australia; 3 Centre for Forensic Behavioural Science, Swinburne University of Technology and Forensicare, Melbourne, VIC Australia; 4 Nossal Institute for Global Health, Melbourne School of Population and Global Health, The University of Melbourne, Melbourne, VIC Australia; 5 Centre for Adolescent Health, Murdoch Children’s Research Institute, The Royal Children’s Hospital Melbourne, Parkville, VIC Australia; 6 Griffith Criminology Institute, Griffith University, Mt Gravatt, QLD Australia; 7 Mater Research Institute-UQ, The University of Queensland, QLD, Brisbane, Australia

**Keywords:** Non-suicidal self-injury, Suicide, Injecting drug use, Prisoner health

## Abstract

**Background:**

Histories of self-harm and suicide attempts are common among people in prison in Australia, and substance dependence is an established risk factor for these lifetime experiences. We describe the prevalence of self-reported history of non-suicidal self-injury (NSSI) and suicide attempts in a cohort of men with recent histories of injecting drug use (IDU) imprisoned in Victoria, Australia. Baseline interviews from the Prison and Transition Health (PATH) prospective cohort study were conducted in the weeks prior to release from prison among 400 incarcerated men who reported regular IDU in the six months prior to incarceration.

**Results:**

Participants completed a researcher-administered structured questionnaire that collected information on sociodemographics, substance use patterns, and physical and mental health indicators. More than one third (37%) reported a history of NSSI and almost half of participants (47%) reported a history of suicide attempts. In multivariable Poisson regression models, a history of NSSI was associated with: being aged 30–39 years; moving accommodation three or more times in the year prior to current sentence; self-reporting a history of mental illness diagnosis; current poor psychiatric well-being; and self-reporting three or more previous drug overdoses. History of suicide attempts was significantly associated with: self-reporting a history of mental illness diagnosis; current poor psychiatric well-being; and self-reporting a history of 1–2 and > 3 drug overdoses.

**Conclusion:**

We observed a disconcertingly high lifetime prevalence of non-suicidal self-injury and suicide attempts among incarcerated males with a history of recent regular injecting drug use. Significant associations with indicators of mental illness and drug related harms support the need to prioritise in-prison screening and early intervention to reduce the risk of future harms for this population.

## Background

People in prison are distinguished by a high prevalence of mental health problems (Chang et al., 2015; Schilders and Ogloff, 2014) which contribute to high rates of self-harm and suicide attempts among people in prison compared to the general community (Borschmann, Thomas, et al., 2017; Fazel et al., 2011; Hawton et al., 2014). Rates of completed suicide among men in prison in Australia have been reported at almost 4 times the rate seen in the general population (Fazel et al., 2011), while almost one quarter of Australian prisoners report a history of self-harm (Australian Institute of Health and Welfare, 2015), compared to approximately 8% of the general population (Moller et al., 2013).

A key contributor to this disproportionate rate of suicide and self-harm in prisoner populations is the strong association among people with a history of injecting drug use (IDU) (Darke & Ross, 2002), a characteristic disproportionately represented among people in prison (Beyrer et al., 2003; Jürgens et al., 2011; World Health Organization, 1994). Almost half of Australian prisoners report a history of IDU (Australian Institute of Health and Welfare, 2015), while over half of people who inject drugs (PWID) sampled in Australian capital cities report previous incarceration (Stafford & Breen, 2017). Suicide rates among PWID have been estimated at 14 times that seen in the general population, while the major risk factors associated with self-harm and suicide including psychological distress, family conflict and social isolation are over-represented among PWID (Darke and Ross, 2002). Additionally, there is some evidence of an association between history of overdose and history of suicide attempts, however, this association remains unclear in the context of accidental overdose and substance misuse (Maloney et al., 2009).

Upon release from prison, people are exposed to many challenges and display a range of vulnerabilities that may increase their risk of self-harm and suicidal behaviour, including feelings of hopelessness (Kariminia et al., 2007), unstable accommodation (Lim et al., 2012), reduced social supports (Jenkins et al., 2005), and marginalisation (Kinner, 2006). The impact of these vulnerabilities is magnified for people in prison with mental illness and dependent substance use co-morbidities. It is well documented that these co-morbidities are associated with increased risk of self-harm and suicide (Beasley, 1999; Haw et al., 2001; Kariminia et al., 2007), emphasising the importance of targeted screening and identifying risk prior to release in prisoners with drug dependence.

Previous studies have investigated self-harm and suicide attempts in people in prison by reporting prevalence and examining risk factors identified within general prison populations (Armstrong et al., 2014; Fazel et al., 2011; Larney et al., 2012). Risk factors for self-harm and suicide for people in prison include mental illness and substance dependence (Appleby et al., 2004; Fruehwald et al., 2004), but despite the putative role of substance dependence histories in these outcomes, no studies have specifically examined self-harm and suicide attempts in a group of prisoners who inject drugs.

In this paper we describe the lifetime prevalence of non-suicidal self-injury (NSSI), defined as the deliberate inflicting of physical pain with no suicidal intent (Wilkinson and Goodyer, 2011), and attempted suicide in a cohort of incarcerated men pending release with a history of recent regular IDU prior to their current sentence. In particular, we explore the characteristics associated with both of these lifetime experiences, with a view to highlight these characteristics in the context of future risk of NSSI and suicide attempt.

## Methods

Data were collected from baseline interviews (*N* = 400) from the Prison and Transition Health (PATH) Cohort Study. PATH is a prospective cohort study that includes three components of data collection, in-depth quantitative interviews, blood sampling and blood borne virus testing, and record linkage to health and justice databases. The study aims to characterise the prison-to-community trajectories of incarcerated males reporting recent regular IDU prior to their sentence and identify factors associated with health, social and crimiogenic outcomes. Women were included in the original study design; however, operational pressures at the main women’s prison in Victoria at the time of study implementation precluded their recruitment.

Baseline interviews were conducted in the weeks preceding release from prison (median = 33 days before release, IQR = 13–62 days) among incarcerated men reporting recent regular IDU prior to their current sentence. Participants were recruited from one minimum, one medium, and one maximum-security correctional facility in Victoria, Australia. Eligible participants (≥18 years of age, IDU reported at least monthly in the six months prior to their current sentence, expected release within six weeks post-interview, and able to provide informed, written consent) completed a researcher-administered structured questionnaire canvassing a range of issues, including sociodemographics, substance use patterns, and physical and mental health indicators. Questionnaire items were modified from the Melbourne Injecting Drug User Cohort Study (Horyniak et al., 2013), a community-recruited cohort of PWID in Melbourne, and incorporated standardised and validated scales to measure current mental health status (Goldberg, 1992) and alcohol use prior to prison (Babor et al., 2001). Interviews occurred between September 2014 and May 2016, and the mean duration of interviews was 43 min (SD = 28 min, range: 23–73 min). Individuals on remand (pre-trial detention) and females were excluded from the study.

Ethics approval for this study was granted by the Alfred Hospital Human Research Ethics Committee (79/12) and the Victorian Department of Justice Human Research Ethics Committee (CF/14/10169).

### Outcome measures

A history of NSSI and suicide attempts was assessed via self-report. Participants were asked, “Have you ever attempted suicide?” and subsequently, “Excluding suicide attempts, have you ever deliberately harmed or injured yourself?” Responses were recorded as yes or no. If concerns about participants’ wellbeing were identified at the time of interview, interviewers were instructed to terminate interviews and refer to prison mental health services. No interviews were terminated on this basis.

### Correlates

Based on previous studies of justice involved populations (Borschmann, Thomas, et al., 2017; Hawton et al., 2014; Larney et al., 2012), a range of potential correlates of history of NSSI and history of suicide attempts were selected from the sociodemographic, general health, mental health, substance use, and criminal justice domains. Aboriginal and Torres Strait Islander status was included as a correlate in the analysis given the over-representation of this group in prison settings in Australia (Australian Bureau of Statistics, 2017), and the high prevalence of self-harm and illicit drug use in this group compared to non-Aboriginal and Torres Strait Islanders Australians (Australian Institute of Health and Welfare, 2014, 2018).

#### Sociodemographics

Age (≤ 29/30–39/≥40 years), number of completed years of education (≤9/≥10 years completed), times moved accommodation in the year before current sentence (none/1–2/≥3 times), employment status prior to current sentence (unemployed or mostly without work/continuous or intermittent employment), relationship status (single/married or regular partner), dependent children with or without Department of Health and Human Services (DHHS) involvement (no children/children without DHHS involvement/children with DHHS involvement), ever removed from family home as a child (yes/no), and Aboriginal and Torres Strait Islander identification (yes/no).

#### General health

General health rating (excellent to good/fair to poor) and self-reported intellectual disability (yes/no).

#### Mental health

Contact with mental health services ever (yes/no), self-reported mental health diagnosis ever (inclusive of only mood, anxiety, psychotic and personality related disorders) (yes/no), and level of psychiatric well-being as assessed using the 12-item General Health Questionnaire (GHQ-12) with the mean-derived cut-off threshold used to identify current poor psychiatric wellbeing (above mean/below mean) (Goldberg et al., 1998).

#### Substance use

Duration of injecting history (median split) (≤18/> 18 years), drug type used in the week prior to their current sentence (heroin only/methamphetamines only/ heroin and methamphetamines/other), number of self-reported drug overdoses ever (none/1–2/≥3), and high risk alcohol intake (at least two times per week and at least five or more drinks per typical drinking episode) in the year prior to current sentence (yes/no).

#### Criminal justice

History of juvenile incarceration (yes/no) and self-reported number of adult incarceration episodes (median split) (≤5/> 5).

### Statistical analysis

Descriptive statistics were generated for each exposure variable and disaggregated by history of NSSI and history of suicide attempts. Exposure measures were examined with Poisson regression using odds ratios (ORs) and 95% confidence intervals (95% CIs). Bivariate analyses were conducted to examine the associations between each potential correlate and history of NSSI and history of suicide attempt. All correlates were entered into separate multivariable models. Thirty-six participants (9%) were excluded from analysis because of missing data. There were no significant differences between participants included in or excluded from analysis. Statistical significance was set at *p* < 0.05. All analyses were conducted using Stata 15.1 for Windows (StataCorp, 2017).

## Results

### Sample characteristics

Most of the 364 participants included for analysis were aged over 30 years (75%), with a median age of 35.6 years (IQR = 11.9). Almost half completed less than 10 years of education (41%), reported limited or no employment prior to their current sentence (44%) and 63% reported being single. Sixteen precent identified as Aboriginal and/or Torres Strait Islander, more than two-thirds had relocated accommodation in the year prior to their current sentence (65%) (a mean 3.2 times), and 25% reported having ever been removed from their family home as a child. Of the 221 men with at least one child, 29% (*n* = 105) reported a history of government involvement in child rearing. A history of NSSI was reported by 133 (37%) men and a history of suicide attempt was reported by 172 (47%) men; 109 (30%) men reported a history of both NSSI and history of suicide attempt (Table 1).

**Table 1:** Baseline characteristics, bivariate associations and modified poisson regression associations for history of non-suicidal self-injury and history of suicide attempt among incarcerated men reporting recent regular injecting drug use prior to current sentence (*n* = 364)

	Total participants	History of non-suicidal self-injury			History of suicide attempts		
	*n* = 364 (%)	*n* = 133 (%)	OR^a^ (95% CI)	aOR^b^ (95% CI)	*n* = 172 (%)	OR^a^ (95% CI)	aOR^b^ (95% CI)
Socio-demographic							
Age							
≤ 29	90 (25)	39 (29)	1.72 (1.16–2.54)*	1.53 (0.91–2.55)	40 (23)	1	1
30–39	155 (42)	64 (48)	1.64 (1.14–2.35)*	1.46 (1.01–2.10)*	78 (45)	1.13 (0.86–1.50)	1.11 (0.82–1.51)
≥ 40	119 (33)	30 (23)	1	1	54 (32)	1.02 (0.75–1.38)	1.19 (0.81–1.75)
Completed years of education							
≤ 9 years	150 (41)	61 (46)	1	1	72 (42)	1	1
≥ 10 years	214 (59)	72 (54)	0.83 (0.63–1.08)	0.88 (0.66–1.19)	100 (58)	0.97 (0.78–1.21)	0.98 (0.77–1.23)
Times moved accommodation^c^							
None	139 (38)	40 (30)	1	1	60 (35)	1	1
1–2	94 (26)	35 (26)	1.29 (0.89–1.88)	1.35 (0.92–1.98)	44 (26)	1.08 (0.81–1.45)	1.16 (0.87–1.53)
≥ 3	131 (36)	58 (44)	1.54 (1.11–2.13)*	1.40 (1.01–1.95)*	48 (39)	1.20 (0.93–1.55)	1.13 (0.88–1.46)
Employment status							
Continuous/intermittent	205 (56)	70 (53)	1	1	95 (55)	1	1
Unemployed/mostly without work	159 (44)	63 (47)	1.16 (0.89–1.52)	1.08 (0.81–1.43)	77 (45)	1.05 (0.84–1.30)	0.96 (0.77–1.21)
Aboriginal and/or Torres Strait Islander							
No	304 (84)	107 (80)	1	1	139 (81)	1	1
Yes	60 (16)	26 (20)	1.23 (0.89–1.71)	1.15 (0.83–1.61)	33 (19)	1.20 (0.93–1.56)	1.14 (0.87–1.50)
Relationship status							
Single	228 (63)	81 (61)	1	1	105 (61)	1	1
Married/regular partner	136 (37)	52 (39)	1.08 (0.82–1.42)	1.06 (0.81–1.39)	67 (39)	1.07 (0.86–1.33)	1.08 (0.87–1.34)
Children with/without DHHS involvement							
No children	143 (39)	50 (38)	1	1	61 (35)	1	1
Children without DHHS involvement	116 (32)	38 (28)	0.94 (0.66–1.32)	0.86 (0.60–1.22)	55 (32)	1.11 (0.85–1.46)	0.99 (0.76–1.30)
Children with DHHS involvement	105 (29)	45 (34)	1.23 (0.89–1.68)	1.08 (0.80–1.47)	56 (33)	1.25 (0.96–1.62)	1.06 (0.82–1.37)
Removed from family as child ever							
No	272 (75)	91 (68)	1	1	121 (70)	1	1
Yes	92 (25)	42 (32)	1.36 (1.03–1.80)*	1.13 (0.86–1.50)	51 (30)	1.25 (0.99–1.56)	1.19 (0.93–1.53)
General health							
General health rating							
Excellent/good	275 (76)	95 (71)	1	1	123 (72)	1	1
Fair/poor	89 (24)	38 (29)	1.24 (0.92–1.65)	1.08 (0.79–1.49)	49 (28)	1.23 (0.98–1.55)	1.05 (0.81–1.36)
Intellectual disability							
No	326 (90)	112 (84)	1	1	149 (87)	1	1
Yes	38 (10)	21 (16)	1.61 (1.16–2.22)*	1.27 (0.86–1.88)	23 (13)	1.32 (1.00–1.76)	1.24 (0.91–1.68)
Mental health							
Visited a mental health service ever							
No	47 (13)	10 (8)	1	1	13 (8)	1	1
Yes	317 (87)	123 (92)	1.82 (1.03–3.22)*	1.47 (0.83–2.60)	159 (92)	1.81 (1.13–2.92)*	1.36 (0.86–2.16)
Self-reported mood, anxiety psychotic & personality disorder							
No	82 (23)	16 (12)	1	1	20 (12)	1	1
Yes	282 (77)	117 (88)	2.13 (1.34–3.37)*	1.65 (1.06–2.58)*	152 (88)	2.21 (1.49–3.29)**	1.80 (1.21–2.69)*
GHQ-12 ≥ 3 (poor psychiatric well-being)							
No	205 (56)	59 (44)	1	1	77 (45)	1	1
Yes	159 (44)	74 (56)	1.62 (1.23–2.12)*	1.40 (1.07–1.85)*	95 (55)	1.59 (1.28–1.98)**	1.39 (1.11–1.74)*
Substance use							
Illicit substances used^d^							
Heroin	62 (17)	20 (15)	1	1	24 (14)	1	1
Methamphetamine	140 (38)	49 (37)	1.24 (0.86–1.77)	0.98 (0.64–1.51)	67 (39)	1.24 (0.86–1.77)	1.26 (0.89–1.79)
Heroin & methamphetamine	116 (32)	47 (35)	1.34 (0.93–1.91)	1.12 (0.74–1.69)	60 (35)	1.34 (0.93–1.91)	1.34 (0.95–1.89)
Other	46 (13)	17 (13)	1.18 (0.76–1.84)	1.30 (0.75–2.24)	21 (12)	1.18 (0.76–1.84)	1.32 (0.86–2.00)
Duration of injecting career							
≤ 18 years	182 (50)	72 (54)	1	1	85 (49)	1	1
> 18 years	182 (50)	61 (46)	0.85 (0.65–1.11)	0.98 (0.67–1.42)	87 (51)	1.02 (0.82–1.27)	0.93 (0.70–1.24)
Number of overdoses							
None	151 (41)	41 (31)	1	1	56 (32)	1	1
1–2	82 (23)	34 (25)	1.53 (1.06–2.20)*	1.43 (0.99–2.07)	44 (26)	1.45 (1.08–1.93)*	1.36 (1.01–1.82)*
≥ 3	131 (36)	58 (44)	1.63 (1.18–2.26)*	1.71 (1.21–2.42)*	72 (42)	1.48 (1.14–1.92)*	1.44 (1.10–1.89)*
High risk alcohol use^c^							
No	265 (73)	97 (73)	1	1	119 (69)	1	1
Yes	99 (27)	36 (27)	1.19 (0.95–1.50)	0.91 (0.67–1.24)	53 (31)	1.19 (0.95–1.50)	1.13 (0.89–1.42)
Criminal justice							
Juvenile incarceration							
No	201 (55)	68 (51)	1	1	96 (56)	1	1
Yes	163 (45)	65 (49)	1.18 (0.90–1.54)	1.01 (0.74–1.38)	76 (44)	0.98 (0.78–1.22)	0.88 (0.69–1.13)
Self-reported adult incarceration episodes							
≤ 5	189 (51)	72 (54)	1	1	87 (51)	1	1
> 5	175 (48)	61 (46)	0.92 (0.70–1.20)	0.82 (0.61–1.11)	85 (49)	1.06 (0.85–1.31)	0.98 (0.79–1.22)

Note: *P* values: * = *p* value < 0.05; ** = *p* value < 0.001; ^a^OR = Odds ratio; ^b^aOR = Adjusted odds ratio; collinearity was tested for, both multivariate models showed VIF < 4; ^c^The twelve months prior to current sentence; ^d^The week prior to current sentence

### Associations with history of non-suicidal self-injury ever

In bivariate analyses, being aged ≤29 years (OR = 1.72; 95% CI = 1.16–2.54) or 30–39 years (OR = 1.64; 95% CI = 1.14–2.35), moving accommodation three or more times in the year prior to their current sentence (OR = 1.54; 95% CI = 1.11–2.13), being removed from the family home as a child ever (OR = 1.36; 95% CI = 1.03–1.80), self-reported intellectual disability (OR = 1.61; 95% CI = 1.16–2.22), contact with a mental health service ever (OR = 1.82; 95% CI = 1.03–3.22), self-reporting a history of mental illness diagnosis (OR = 2.13; 95% CI = 1.34–3.37), current poor psychiatric well-being (OR = 1.62; 95% CI = 1.23–2.12), and self-reporting 1–2 (OR = 1.53; 95% CI = 1.06–2.20) or ≥ 3 (OR = 1.63; 95% CI = 1.18–2.26) drug overdoses were associated with reporting a history of NSSI.

In multivariate analysis, being aged 30–39 years (adjusted odds ratio [aOR] = 1.46; 95% CI = 1.01–2.10), moving accommodation three or more times in year prior to their current sentence (aOR = 1.40; 95% CI = 1.01–1.95), self-reporting a history of mental illness diagnosis (aOR = 1.65; 95% CI = 1.06–2.58), current poor psychiatric well-being (aOR = 1.40; 95% CI = 1.07–1.85), and self-reporting ≥3 drug overdoses (aOR = 1.71; 95% CI = 1.21–2.42), remained statistically significantly associated with reporting a history of NSSI.

### Associations with history of suicide attempt ever

In bivariate analyses, contact with a mental health service ever (OR = 1.81; 95% CI = 1.13–2.92), self-reporting a history of mental illness diagnosis (OR = 2.21; 95% CI = 1.49–3.29), current poor psychiatric well-being (OR = 1.59; 95% CI = 1.28–1.98), and self-reporting 1–2 (OR = 1.45; 95% CI = 1.08–1.93) or ≥ 3 (OR = 1.48; 95% CI = 1.14–1.92) drug overdoses ever were associated with reporting a history of suicide attempt.

In multivariate analysis, self-reporting a history of mental illness diagnosis (aOR = 1.80; 95% CI = 1.21–2.69); current poor psychiatric well-being (aOR = 1.39; 95% CI = 1.11–1.74), and self-reporting 1–2 (aOR = 1.36; 95% CI = 1.01–1.82) and ≥ 3 (aOR = 1.44; 95% CI = 1.10–1.89) drug overdoses ever, remained statistically significantly associated with reporting a history of suicide attempt.

## Discussion

In this study, we explored the characteristics associated with history of NSSI and history of suicide attempts in a sample of incarcerated men in Australia reporting a history of recent regular IDU prior to their current sentence. Previous studies have highlighted the contribution of substance use (Borges et al., 2000), and IDU (Artenie et al., 2015), to risk of NSSI and suicide attempts among people in prison. However, to our knowledge this is the first estimate of the prevalence of these outcomes in a sample of men in prison with histories of IDU. More than one third of the participants self-reported a history of NSSI and almost half self-reported a history of suicide attempt; this compares to one quarter of the general prison population reporting a history of intentional self-harm in Australia (Australian Institute of Health and Welfare, 2015). Consistent with findings from studies of general prison populations, we found participants self-reporting any history of mental illness diagnosis (Blaauw et al., 2005; Borschmann, Thomas, et al., 2017), poor current psychiatric wellbeing (Dear et al., 2001) and a history of drug overdose (Maloney et al., 2009) were more likely to report a history of NSSI and/or suicide attempt. Additionally, we found that people in prison aged between 30 and 39 years were more likely to have a history of NSSI compared to those aged over 40, which is consistent with general population data (Harrison and Henley, 2014).

History of self-harm (Cooper et al., 2005), history of suicide attempts and prior or current mental illness are the most robust clinical predictors for subsequent suicide attempt or completed suicide (Bostwick et al., 2016; Steele et al., 2017). Our findings of an association between NSSI and suicide attempt, and indicators of mental illness and current psychiatric morbidity in this cohort of soon to be released prisoners provides useful markers of potential future risk and can inform early opportunities to intervene. Prison reception has been identified as an opportunity to identify people with serious mental illness who may be at risk of self-harm (Ogloff et al., 2007). Almost half (47%) of male prisoners in Australia report a history of mental health problems (Australian Institute of Health and Welfare, 2015), consistent with findings elsewhere (Fazel and Seewald, 2012). To respond to this high prevalence within an often-constrained resource environment, identifying people in prison requiring the most immediate need is paramount. However, previous research has reported a fragmented approach to mental health screening in prison across Australian jurisdictions (Ogloff et al., 2007). While in-depth screening is unfeasible given time and resource constraints, brief validated screening tools such as the Jail Screening Assessment Tool (JSAT), which do not require clinical expertise and include screening for risk of self-harm and suicide (Nicholls et al., 2005), offer a potentially valuable mechanism for referral and follow-up. However, referrals to additional services following the screening of acute mental illness at prison reception has been shown to be vastly under-utilised (Schilders and Ogloff, 2014). In this context, the correlates of NSSI and suicide attempts reported in this paper could be used to either target the use of screening tools or used alongside screening tools to prioritise referral.

Community health service contacts following prison release also offer an opportunity to identify those at risk of self-harm and suicide. Past contact with mental health services and/or history of mental illness diagnosis was significantly associated with history of NSSI and suicide attempts, while  87% of prisoners in the study reported prior contact with mental health services. Intervention opportunities are, however, not restricted to specialist mental health services. Australian research exploring the use of tertiary health services among PWID found PWID to be frequent users of emergency department (ED) and tertiary healthcare services and that ED presentations and hospital separations were most commonly related to mental and behavioural disorders (Nambiar et al., 2018; Nambiar et al., 2017). Similarly, a study of ambulance attendances among recently released prisoners found that one in twelve attendances involved self-harm or self-harm/suicidal ideation (Borschmann, Young, et al., 2017). Another Australian study found almost half of participants presenting to ED for self-harm following release from prison were previously identified in prison health records as being at risk of self-harm (Borschmann, Thomas, et al., 2017). These findings suggest the need to better utilise the often frequent contacts that occur between prison health, mental health and tertiary care providers among those at high risk of NSSI and suicide attempts. The strengthening of systems to effectively share clinical and other risk information between services is crucial, alongside the strengthening of effective and enduring referrals pathways.

Our finding that history of drug overdose was associated with both NSSI and suicide attempts is consistent with previous research (Darke et al., 2005; Maloney et al., 2009; Rossow & Lauritzen, 1999). The overlap of suicide attempt and history of drug overdose has been explored to determine the extent to which these lifetime experiences are related (Bohnert et al., 2010; Maloney et al., 2009; Rossow and Lauritzen, 1999). While concluding that opioid dependence was not independently associated with suicide attempts, a case-control study did however find that almost one in five serious suicide attempts among those classified as opioid dependent involved heroin overdose, whereas no serious suicide attempts involved heroin among non-opioid dependent individuals (Maloney et al., 2007). It is nevertheless difficult to determine the degree of deliberate intention to die in cases of fatal drug overdose, given that the risks associated with overdose when injecting opioids have been described as “balancing on the edge of death” (Rossow and Lauritzen, 1999). Collectively, our findings and the findings of previous studies suggest that people self-reporting both history of suicide attempt and drug overdose represent a cohort of people experiencing both poor psychiatric well-being and substance dependence (Bohnert et al., 2010; Maloney et al., 2009; Rossow and Lauritzen, 1999). Further research is needed to better understand the overlap of these lifetime experiences and to determine whether current interventions to reduce the risk of subsequent suicide attempts and/or drug overdoses are effective.

Our study concluded that people in prison identifying as Aboriginal and/or Torres Strait Islander were no more likely than their non-Aboriginal and/or Torres Strait Islander counterparts to report history of NSSI or suicide attempts, this was consistent with previous research findings (Butler et al., 2007; Kariminia et al., 2007; Spittal et al., 2014). Nevertheless, this does contrast with some studies reporting an association between suicide attempt and Aboriginal and/or Torres Strait Islander identification among people in prison (Larney et al., 2012; Stewart et al., 2004). This discrepancy may be explained by the variance in study samples and methods; telephone and face-to face interviewing may exert disparate influence on participant responses. In addition, study location could alter the context of the results in relation to the representation of Aboriginal and/or Torres Strait Islander peoples in correctional settings. Studies recording associations between suicide attempt and Aboriginal and/or Torres Strait Islander identification were undertaken in New South Wales and Western Australia, states with relatively higher rates of Aboriginal and/or Torres Strait Islander prison entrants (43% and 45%, respectively, in 2015) compared to Victoria (11%) (Australian Institute of Health and Welfare, 2015). Studies reporting significant findings for Aboriginal and/or Torres Strait Islander identification were also inclusive of the broader prison population, including those without a history of recent regular IDU (Larney et al., 2012; Stewart et al., 2004). Our study, however, was focused upon men in prison with histories of recent regular IDU, therefore, it is possible that the elevated risk associated with IDU, co-occurring disadvantage and co-morbidity was dominant relative to specific risks among Aboriginal and/or Torres Strait Islander participants.

Our data were collected via self-report making results susceptible to reporting and recall bias, particularly in relation to survey questions exploring pre-incarceration behaviours. Social desirability bias may emerge in relation to certain behaviours resulting in over- or under-reporting of experiences (Darke, 1998). However, self-report among PWID has previously been considered sufficiently reliable in collecting information on drug use behaviours (Darke, 1998). Due to the inability to implement random sampling or extend the study to include incarcerated women or individuals on remand, our results may not be generalizable to the broader prison population, nor reflect the risk of NSSI and suicide attempt among women in prison or individuals on remand. Finally, as this is a cross-sectional analysis, we were unable to establish temporality between correlates and outcomes; this will be important to analyse with prospective data collection. As the PATH cohort study continues to collect post-release follow-up data and the addition of future record linkage, the methodology will allow us to prospectively examine the incidence and correlates of self-harm post release from prison. We can also determine if the risk factors evident in this paper are consistent with those that predict future incidence of self-harm and suicide attempts.

## Conclusion

To the best of our knowledge this is the first study to report on the characteristics associated with NSSI and suicide attempts among incarcerated males reporting recent regular IDU prior to their current sentence. Our findings of the association between history of mental illness and history of drug overdose and reporting a past history of NSSI or suicide attempts can help inform targeted risk screening at the point of prison reception. In addition, community health service contacts by this population provide an opportunity for early intervention, while information sharing between health and justice services could assist in highlighting those at greatest need of intervention to prevent future risk of self-harm and suicide attempts.

## Abbreviations

aOR: Adjusted odds ratio
DHHS: Department of Health and Human Services
IDU: Injecting drug use
NSSI: Non-suicidal self-injury
OR: Odds ratio
PATH: Prison and Transition Health
PWDI: People who inject drugs
